# Northern Limits of Milk-sickness

**Published:** 1843-01

**Authors:** 


### NORTHERN LIMITS OF MILK-SICKNESS.

We have not been able to hear of this disease further north than the southern shore of Lake Erie, between Cleveland and Sandusky city, in N. lat. 41° 25′. On various occasions, we have expressed the belief, that popular opinion, both in and out of the profession, has greatly exaggerated the frequency and importance of this *alledged* specific disease, and our late researches have afforded us no reason for retracting what we have said. After spending several weeks in regions of country reputed to be obnoxious to the malady, we left them without seeing a single case, although the period of the year was that of its recurrence. D.

